# Upper lip malignant neoplasms. A study of 59 cases

**DOI:** 10.4317/medoral.17501

**Published:** 2011-12-06

**Authors:** Kuauhyama Luna-Ortiz, Agustín Güemes-Meza, Verónica Villavicencio-Valencia, Adalberto Mosqueda-Taylor

**Affiliations:** 1 Department of Head and Neck Surgery, Instituto Nacional de Cancerología, México; 2 Professor of Surgical Oncology, Faculty of Medicine, Universidad Nacional Autónoma de México; 3 Professor of Oral Pathology and Medicine. Department of Health Care. Universidad Autónoma Metropolitana Xochimilco. México, D.F

## Abstract

Objectives: To present the demographic data, clinico-pathologic features and therapeutic outcome of a series of upper lip malignancies. Study Design: Retrospective study at a single Cancer Institution in Mexico City during a 14-year period. Results: There were 59 cases, (30 males and 29 females); age range: 14 to 106 years (mean: 73 yr.). Antecedents of ultraviolet light and tobacco exposure were found in 20 (33.9%) and 16 cases (27%) respectively. There were 35 squamous cell carcinomas (59.3%), 19 basal cell carcinomas (32.2%) and one case each (1.7%) of adenocarcinoma NOS, adenoid cystic carcinoma, angiosarcoma, Merkel cell carcinoma and melanoma. There were 14 cases in stage I (23.7%), 14 in stage II (23.7%), 3 in stage III (5.1%) 14 in stage IV (23.7%) and 14 were not classified (23.7%). There were no significant differences with respect to the overall survival curve and the disease-free survival curve among surgical treatment and radiotherapy. In addition, there was not statistically significant difference in the overall survival and disease-free survival among squamous cell carcinoma and basal cell carcinoma cases with respect to the type of treatment. Conclusions: Upper lip malignant neoplasms are infrequent lesions. The present series describes the main clinico-pathological features in a hospital-based population in Mexico city and demonstrates some differences with respect to those found in the lower lip.

** Key words:**Upper lip, neoplasms, basal cell carcinoma, squamous cell carcinoma, melanoma.

## Introduction

Malignant neoplasms of the upper lip are relatively infrequent lesions as compared to those found in the lower lip (1-3). Although squamous cell carcinoma (SCC) is the most frequent cancer of the lower lip and show a male predominance, basal cell carcinoma (BCC) tend to be most frequent in the upper lip, and in this location cancer incidence is similar for both genders or it is even higher in female patients (4).

The aim of this study is to present the demographic data, clinico-pathologic features and therapeutic outcome of the upper lip malignant neoplasms diagnosed in a single Cancer Institution in Mexico City during an 11-year period.

## Material and Methods

We reviewed the clinical records of the patients with upper lip cancer that were admitted at the Instituto Nacional de Cancerología (México) between 1990 and 2000. Demographic data included age, gender, type of occupation (related or not to ultraviolet light exposure), and tobacco smoking antecedents. In addition, relevant data about time of evolution, histopathological diagnosis, type of treatment, recurrence or persistence of the disease, follow-up, and current status of each patient were recorded. Both primary and recurrent lesions that developed in the upper lip were included in this study. We accepted as recurrence those lesions that presented the same histology as the original tumor and involved the primary site or regional lymph nodes and were detected after 6 months of having completed the treatment; the presence of the disease before this period was considered persistence. Likewise, we recorded the secondary treatment performed in each case.

The disease was classified according to its histology. In cases of squamous cell carcinoma the grading system of the classification proposed by Broders (5) was used. Clinical stage was determined according to the American Joint Committee on Cancer (AJCC), and location of the tumor was stated as upper lip; the lip was in turn divided in three sub-sites or thirds: central, left and right, according to the site of origin of the tumor. The presence of regional lymph node involvement, as well as distant metastases both at the time of admittance and during the course of treatment were recorded.

Statistical evaluation was performed by means of Chi-square and Student’s t tests, and those statistically significant differences were subjected to Cox multivariate analysis. Survival was assessed through the Kaplan and Meier curve. All tests were assessed at a confidence interval of 95%, and statistical significance was set at p<0.05.

## Results

**Figure 1:**
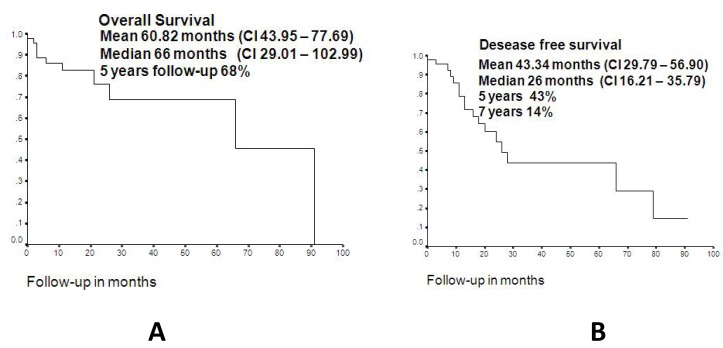
A. Overall survival curve of the present series. B. Disease-free period of the present series.

**Figure 2:**
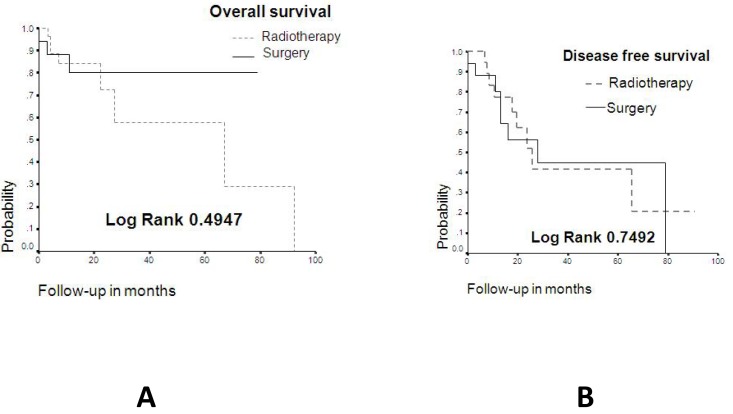
A. Overall survival curve according to primary treatment, B. Disease free period according to primary treatment.

There were 59 cases of upper lip malignancies. Of these, 30 (50.8%) occurred in males and 29 (49.2%) in females, with an age range from 14 to 106 years (mean: 73 yr.). The salient clinical and demographic features are shown in ( Table 1). Histopathologic diagnosis and histological grading are shown in ( Table 2), where it is shown that although SCC and BCC carcinomas accounted for more than 90% of the cases, there were five other different malignant neoplasms diagnosed. Only 46 patients were treated, and there were 4 recurrences (8.7%) and 13 persistent cases (28.3%), comprising a total of 17 cases (36.9%). Comparison among surgical treatment vs radiotherapy is shown in ( Table 3). (Fig. 1) illustrates the overall survival curve (A) and the disease-free period (B). (Fig. 2) show the overall survival curve (A) and the disease free period (B) according to primary treatment, and it was found that there were no significant differences with respect to the overall survival curve and the disease-free survival curve among both types of treatment (p=0.4947 and p=0.7492 respectively). In addition, there was not statistically significant difference in the overall survival (p=0.66) and disease-free survival (p=0.50) among squamous cell carcinoma and basal cell carcinoma cases with respect to the type of treatment (p=0.347).

**Table 1:**
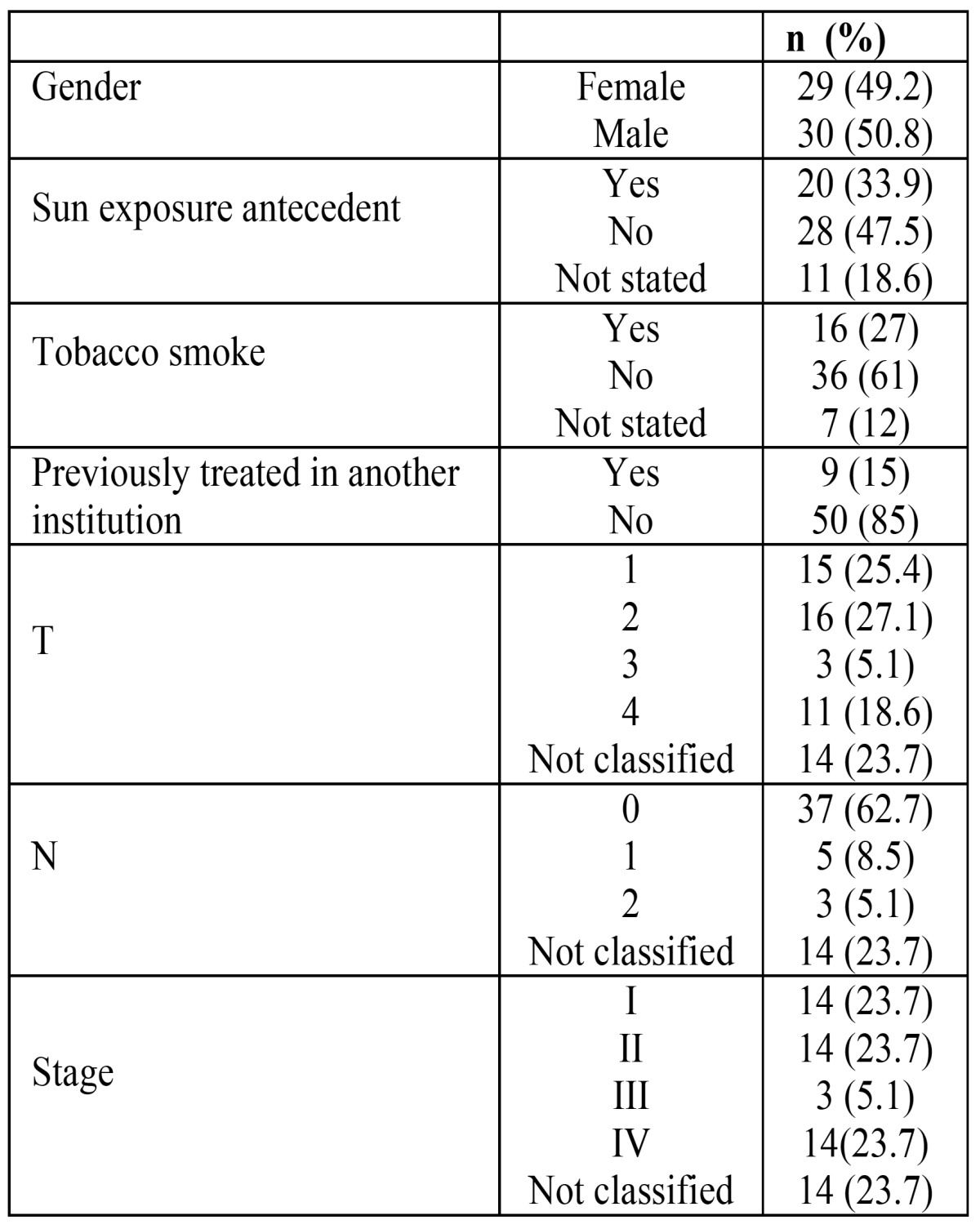
Clinical and demographic features of upper lip malignancies in the present series.

**Table 2:**
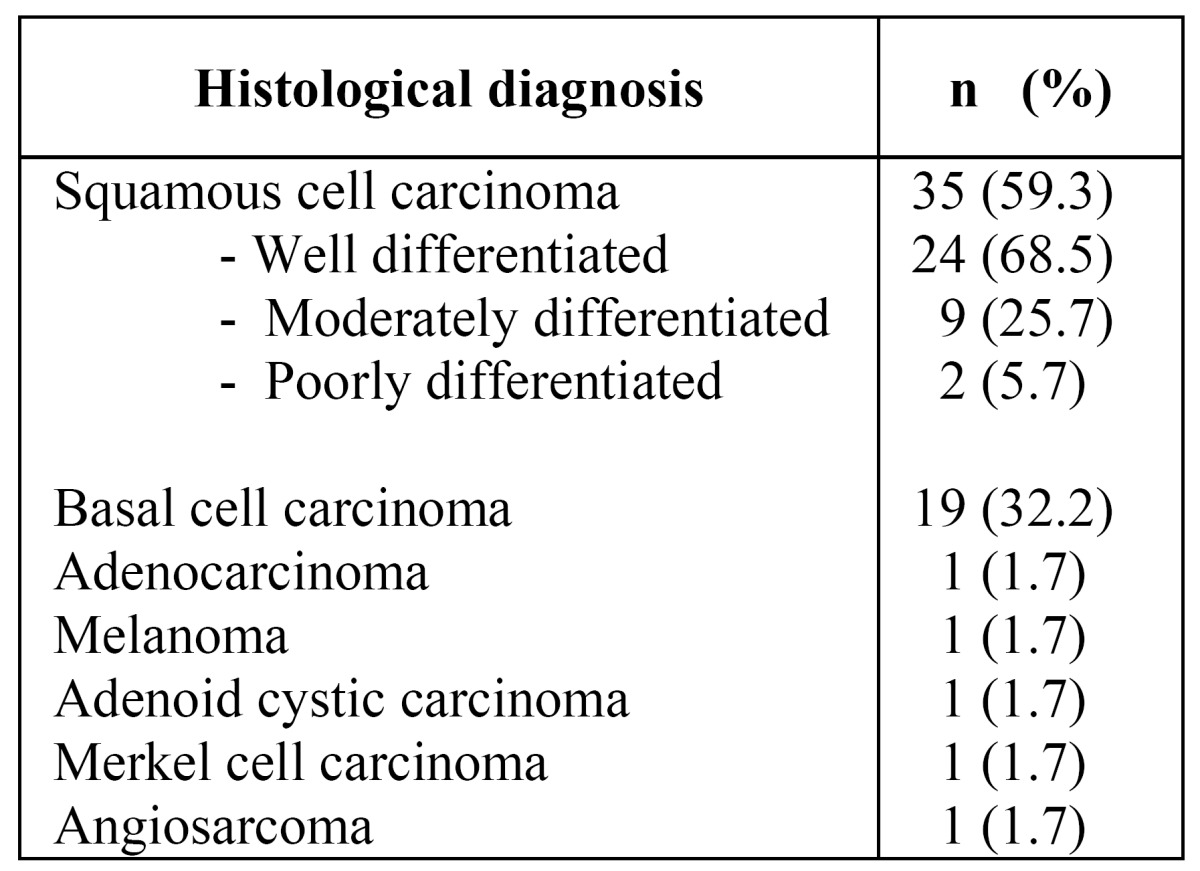
Histopathologic features of upper lip malignancies in the present series.

**Table 3:**
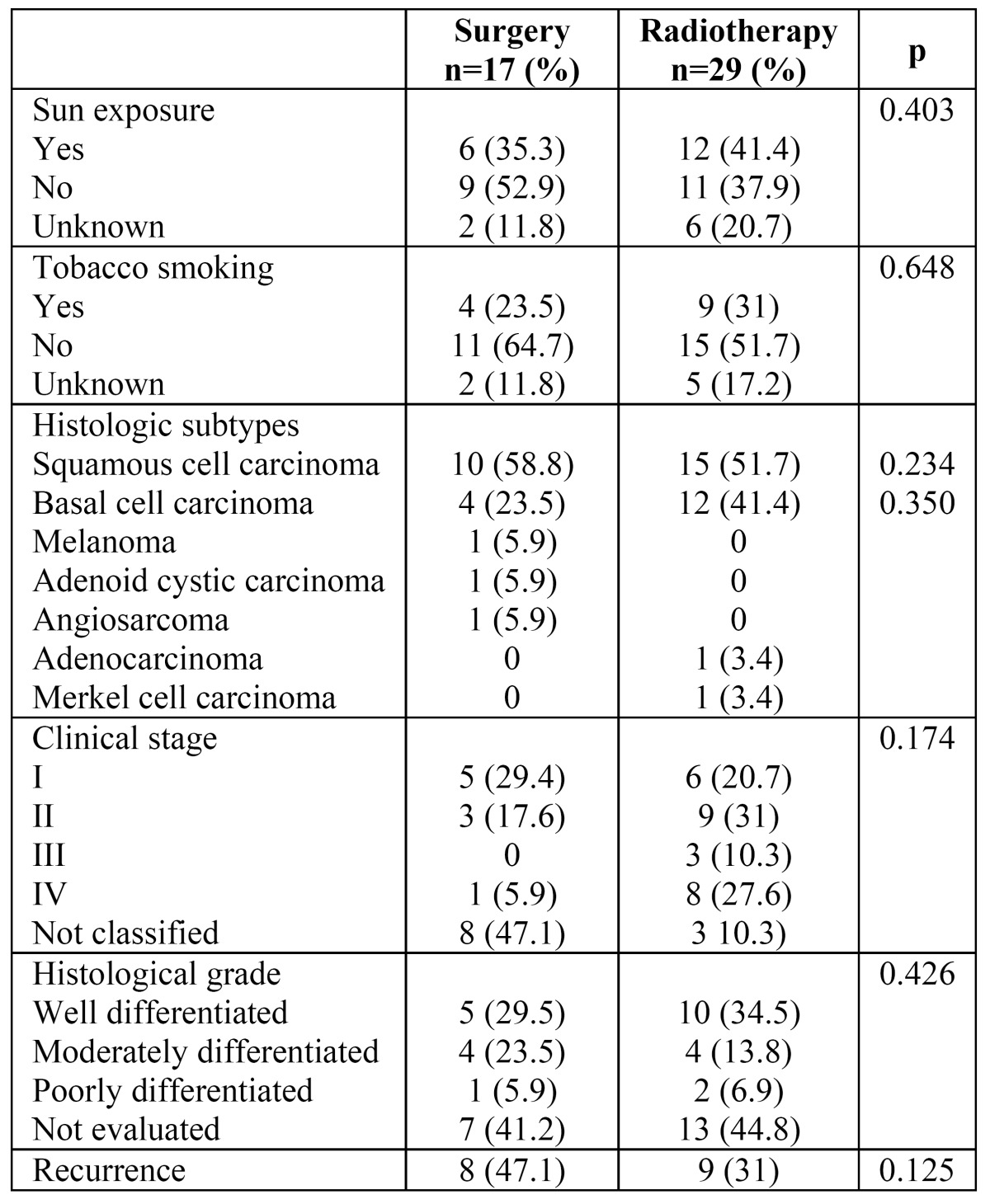
Distribution according to treatment modalities.

(Fig. 3) illustrate two cases in which there is some difficulty to define the exact anatomical site of origin (skin or mucosa) of the upper lip basal cell carcinoma, which usually is not a problem in the case of lower lip SCC.

**Figure 3:**
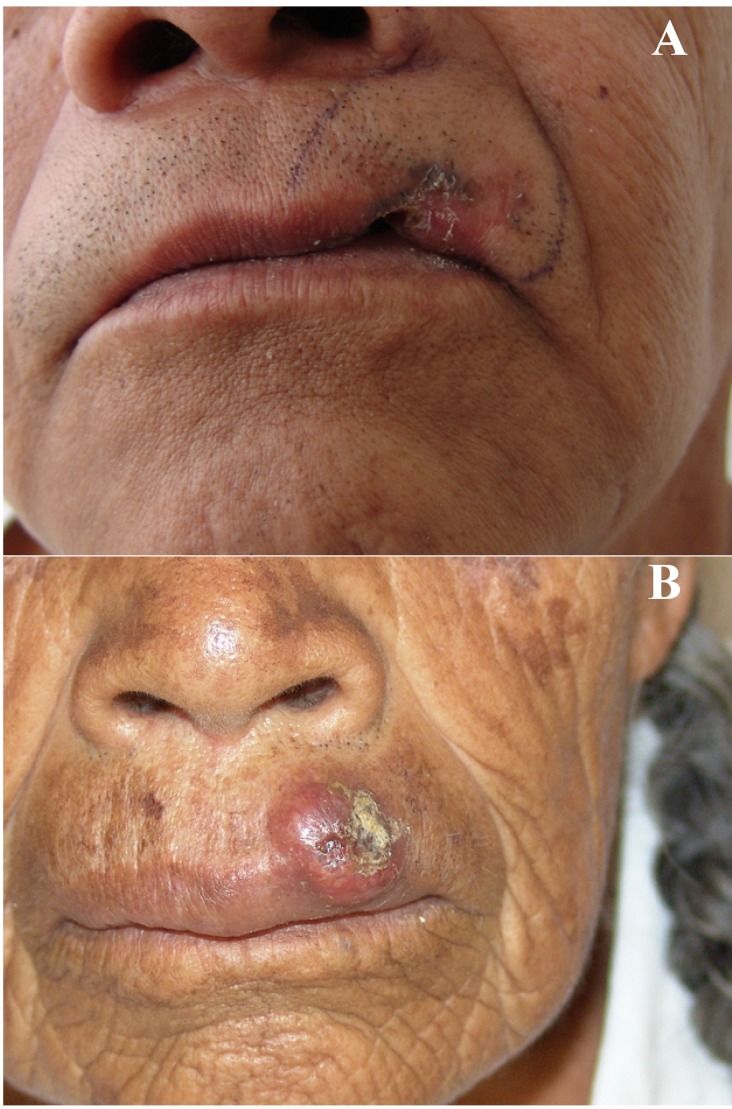
A. Advanced BCC of the upper lip in a 55 year-old male patient in which the site of origin cannot be determined. B. Primary cutaneous BCC with early invasion to the vermilion border in a 66 year-old female patient.

## Discussion

The site of origin of lip tumors seems to be related to their histologic subtype and possible etiological factors, as it has been found that SCC is the most common malignant neoplasm of the lower lip, which is more susceptible to sun-induced carcinogenic damage, while BCC predominates in the upper lip, usually as a consequence of penetration from a neoplasm originated in the adjacent skin epithelium; there are also differences between gender distribution with respect to location, some authors have pointed out that BCC of the upper lip has a predilection to occur in females (4,6-8). In one Canadian study it has been reported that females, particularly young women outnumbered males with basal cell carcinoma of the upper lip, which contrasts with BCC´s diagnosed in other sites in the same population, in which males outnumber females (4). In this study, there were an almost equal number of upper lip malignancies in both genders, with a slight male predominance, which may be explained by the fact that the majority of tumors were SCC´s (59.3%) and that most patients affected by SCC had a positive antecedent of sun exposure (33.9%) and tobacco consumption (27%), which are the most important etiologic factors implicated in the origin of this neoplasm, and they were more frequent in males in the present series, although there were not significant differences for sun exposure and tobacco consumption respectively. Although the possible reason of the higher susceptibility of the lower lip to SCC in older age may lie in the fact that during the aging process the intermaxillary distance is progressively reduced as a consequence of teeth wear, resulting in a protrusion of the lower vermilion border, which is therefore more susceptible to the action of these carcinogens, this explanation is not valid for SCC of the upper lip. There was however, a significant number of patients that consumed tobacco in this group, which may be an important carcinogenic agent to be considered in these cases.

Some studies have suggested that upper lip malignant epithelial neoplasms could derive from pluripotential epithelial cells of the oral mucosa and epidermis (8). Other authors consider their origin from ectopic sebaceous glands (9). On this respect, the incidence of Fordyce granules (ectopic sebaceous glands) tend to increase with advancing age, and these are normally detected in the lips and oral mucosa in a high percentage of senior adults in Western populations (10); however, primary malignant sebaceous tumors in these regions are exceedingly rare (11). Against the hypothesis of an origin of BCC from these glands is the fact that Fordyce granules are more frequent in males (12), contrary to the reported higher incidence of BCC seen in female patients in some series. The other possibility of origin may be from traumatic epithelial implantation (13). To date, there is not a satisfactory explanation about the possible etiologic factors implicated in the origin of BCC of the vermillion and mucosal surfaces of the lips, except for those cases that secondarily invade these structures from adjacent skin.

An important question here is to know if the site of origin of BCC of the lip, that is the vermillion or the skin surface, makes these neoplasms to be considered as different entities; that is, if they should be considered as cutaneous or as oral malignancies, and if such a difference is a factor that determine their different biological behavior, as it has long been known that once a malignant neoplasm extends into the vermillion or the labial mucosa surface, particularly in the case of BCC, acquires a more aggressive biological behavior than its cutaneous counterpart (Fig. 3). From an oncological point of view, we may consider that this separation is not very important, as all malignant epithelial neoplasms, when located in the lip (vermillion or mucosa), even in their earlier stages, tend to invade deep structures, and here tumoral invasion is particularly favored by the fact that the lamina propria in these anatomical regions is very thin and allows the neoplasm to invade nerves and muscles more easily than when the neoplasm is located only in the skin, which is a more resistant structure to deep neoplastic invasion (8). These facts are important from the surgical and functional points of view, as they determine the extent and type of complex reconstructive procedures in this anatomical region.

In our series, 28.8% of the tumors were in advanced stages, which is similar to the findings reported by Zitsch et al. (3), who found that 25% of their cases located in the upper lip were tumors larger than 3 cm, but differ from those reported by Leibovitch et al. (6), who found that 90% of their cases were smaller than 2 cm. In the present study it was found that most patients were of low socioeconomic level and the lack of symptomatology of the neoplasms in their early stages was a definitive factor that caused a delayed diagnosis and hence most cases were treated in advanced stages. This situation is reflected also in our series in that there were 4 (8.6%) recurrences and 13 (28.2%) cases that showed persistence of the neoplasms, which had to be submitted to a new treatment, increasing in this way the functional alterations and the aesthetic aspect of the affected individuals.

It is interesting to note that in this study, in addition to SCC and BCC there were five (8.5%) cases of other malignant neoplasms. To our knowledge, only isolated case reports of these rare tumors affecting the lips have been published, and therefore this study may be important to determine the relative frequency of these unusual malignancies as their management should be individualized according to their specific biological behavior.

In addition, it has to be considered that although there are few histologic subtypes of malignant neoplasms that tend to affect the upper lip, once cutaneous malignancies reaches the upper lip vermillion border or adjacent mucosa, they should be considered a particular type of lip neoplasm, as the prognosis is worst in comparison to those limited to the adjacent skin. There seems to be no differences in terms of survival between cases treated with surgery and those with radiotherapy; however, from a functional point of view this could be different in cases requiring large resections, and therefore surgery is problably best indicated in early (small) lesions.

In conclusion, upper lip malignant neoplasms are infrequent lesions, the present series describes the main clinico-pathological features in a hospital-based population in Mexico city and demonstrates some differences with respect to those found in the lower lip.
